# Supplementary material for: First Experiences with Ultrasound-Guided Transthoracic Needle Biopsy of Small Pulmonary Nodules Using One-Lung Flooding: A Brief Report
Source: Diagnostics (Basel). 2025 Sep 18;15(18):2374. doi: 10.3390/diagnostics15182374 (PMC12468551; doi:10.3390/diagnostics15182374)
Supplement: Supplementary file 1 [file diagnostics-15-02374-s001.zip › diagnostics-3737617-supplementary.pdf]

Supplementary File S1

Method

General procedure

Patients were anesthetized and intubated with a left-sided double-lumen endobronchial tube (DLT) followed by a total intravenous anesthesia and a pressure-controlled ventilation of both lungs. Invasive arterial blood pressure, electrocardiography and transcutaneous oxygen saturation (tcSO2) were monitored throughout the procedure (Datex AS/3, Datex-Ohmeda, Helsinki, Finland). A cuff controller (VBM Medizintechnik GmbH, Sulz a.N., Germany) was used to maintain a constant pressure of 50 cm H2O within both the endobronchial and tracheal cuffs. Immediately before OLF, the patients underwent two-lung ventilation with a fraction of inspired oxygen (FiO2) of 1.0 for 20 min to denitrogenate the lungs. The patients were then placed in the right lateral decubitus position with the lung flooded in the dependent position, and the right lumen of the DLT disconnected from the ventilator. An infusion system was immediately connected to the right limb of the DLT, and the right lung was slowly filled (single filling) with degassed, warmed (37 °C) isotonic saline flowing passively from an infusion bottle suspended 30 cm above heart level (**Supplementary Figure S1**). The volume to be infused was estimated as one-half the functional residual capacity (FRC) of the lungs. Complete saline filling was monitored with transcutaneous lung ultrasound (bk5000; bK medical GmbH, Quickborn, Germany). One-lung ventilation of the non-flooded left lung was performed without changing the setting but decreasing the FiO2 to 0.6. Throughout the procedure the correct position of the DLT was confirmed using a fiber optic. After the right lung could be imaged with ultrasound the patients were placed in the supine position. To avoid dislocation of the DLT during position changing the action was monitored by fiberbronchoscopy. Following location of the pulmonary nodule a biopsy needle was inserted under sonografic guidance and a core biopsy was performed. The speciman was examined by frozen section. After that the patients were placed in trendelenburg position to improve the passive flow out of the fluid through the opened right tubus leg. At the end of drainage the tubus leg was connected with the ventilator and both lung were ventilated. After sufficient spontaneous breathing with an tcSO2 above 90%, the patients were extubated. The patients received alternating oxygen mask (4 liters/min) and continuous positive airway pressure (CPAP) and were monitored initially in the recovery room and later in the intermediate care. 2 and 4 hours after extubation a lung sonography was performed. After 6 hours a chest X-ray was performed.

In case of malignancy the tumour was resected by videothoracoscopy or thoracotomy in the same session. If there was a benigne diagnosis the patients were extubated.

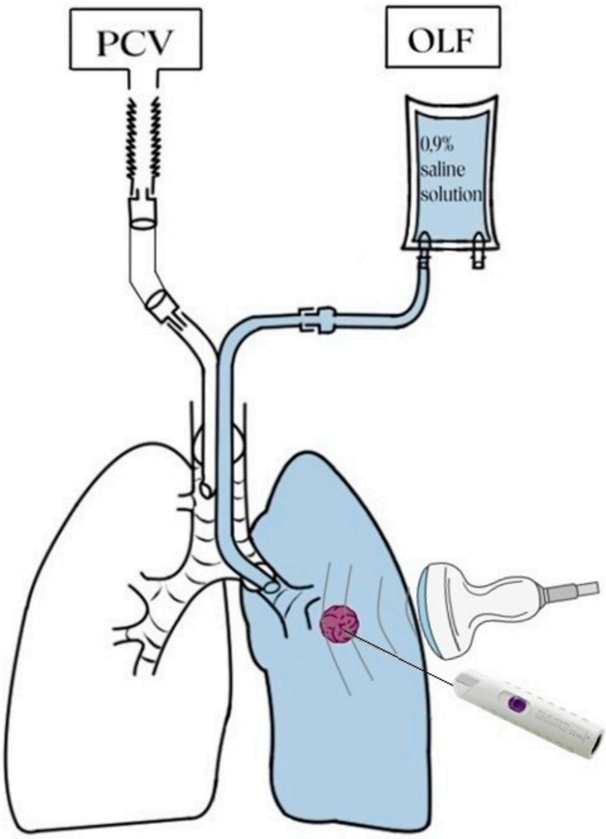

Figure S1. Schema of OLF of the left lung after DLT intubation with one-lung ventilation (right) and ultrasound-guided transthoracic biopsy of a pulmonary nodule.

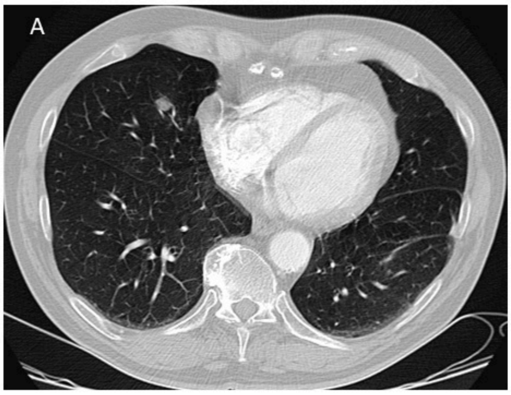

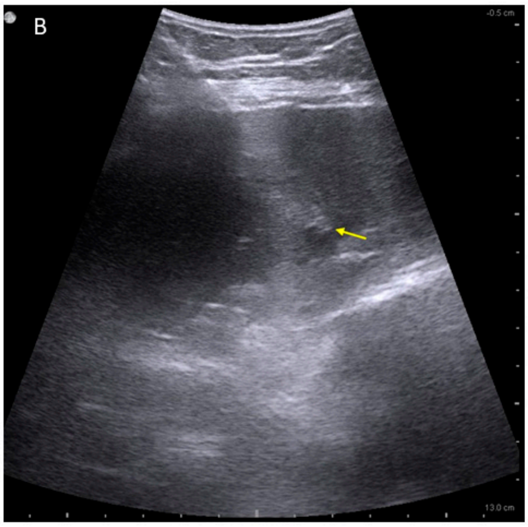

Figure S2. (*A*) CT-scan demonstrated a pulmonary nodule within the middle lobe, 10 mm in diameter and surrounded by vessels. *(B)* Tranthoracic ultrasound of the right lung during OLF. A 10 mm hypoechoic, unsharp marginated nodule, that located about 4 cm below the pleura was puncted. The tip of the core needle (14G) was identified within the nodule (arrow).

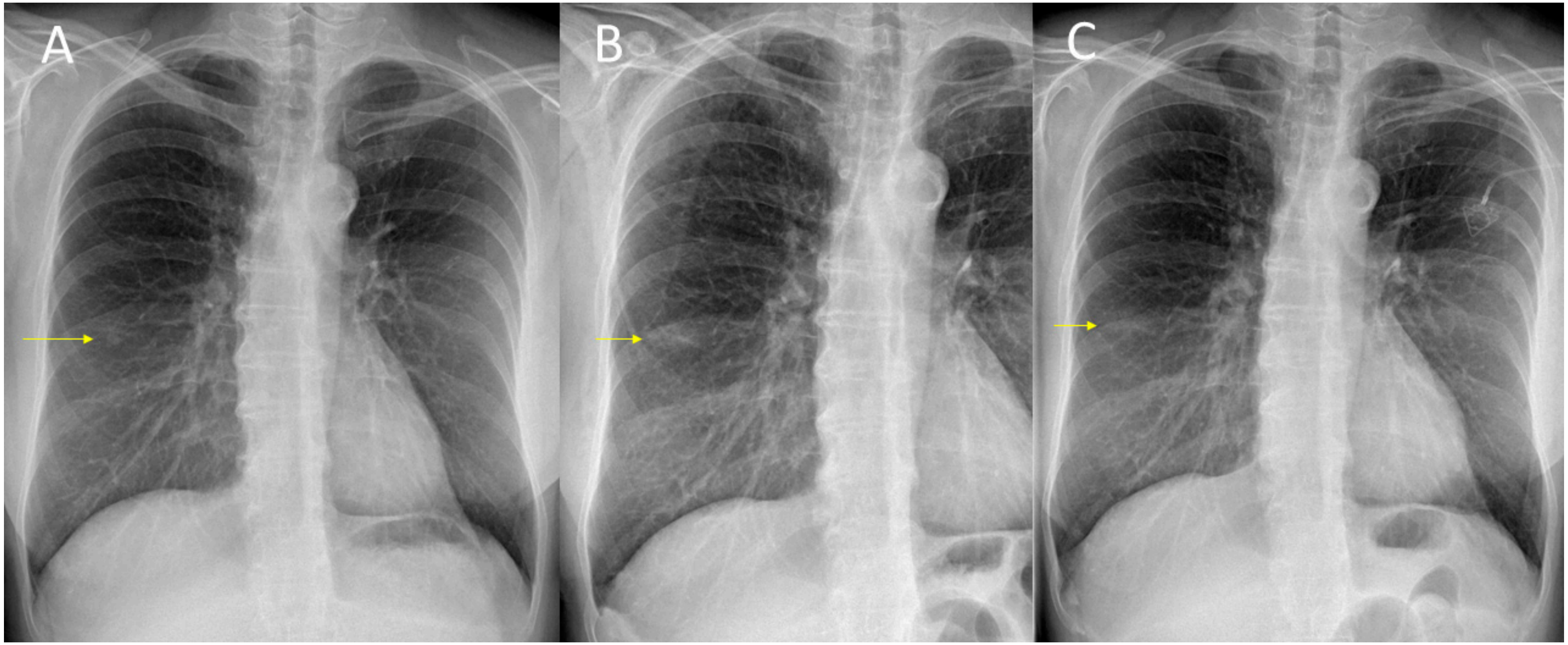

Figure S3. *(A)* Pre-interventional X-ray shows a small nodule within the right lung (arrow). *(B)* X-ray on day two post-interventional shows a very slight perinodular hemorrhage (arrow); no evidence of pneumothorax, pleural effusion and residual lung water. *(C)* X-ray on day nine post-interventional revealed a normal aerated lung with almost completely absorbed hemorrhage.
